# Hairy silica nanosphere supported metal nanoparticles for reductive degradation of dye pollutants†

**DOI:** 10.1039/d1na00020a

**Published:** 2021-03-22

**Authors:** Xin Chen, Li Zhang, Bin Xu, Tingting Chen, Lianhong Hu, Wei Yao, Mengxiang Zhou, Hui Xu

**Affiliations:** Institute of Advanced Synthesis, School of Chemistry and Molecular Engineering, Jiangsu National Synergetic Innovation Center for Advanced Materials, Nanjing Tech University Nanjing 211816 China ias_hxu@njtech.edu.cn; Nanjing Institute of Environmental Sciences, Ministry of Ecology and Environment of the People's Republic of China Nanjing 210042 China

## Abstract

Hairy materials can act as a sort of scaffold for the fabrication of functional hybrid composites. In this work, silica nanospheres modified with covalently grafted poly(4-vinylpyridine) (P4VP) brushes, namely, “hairy” silica spheres, were utilized as a support for the anchorage of metal nanoparticles (MNPs), thus resulting in the hierarchical SiO_2_@P4VP/MNP structure. In this triple-phase boundary heteronanostructure, the SiO_2_-supported MNPs are well stabilized by the P4VP matrix to avoid aggregation and leaching. These SiO_2_@P4VP/MNP nanocomposites exhibit good catalytic activity in the reductive degradation of organic dyes, *i.e.*, 4-nitrophenol and rhodamine B and possess excellent stability and recyclability for five successive cycles.

## Abstract 2

Highly dispersed and tiny-sized noble metal nanoparticles are deposited on a hairy poly(4-vinylpyridine)-silica support, which can serve as recyclable heterogeneous catalysts in the reductive degradation of 4-NP and RhB dye pollutants.

## Graphical abstract

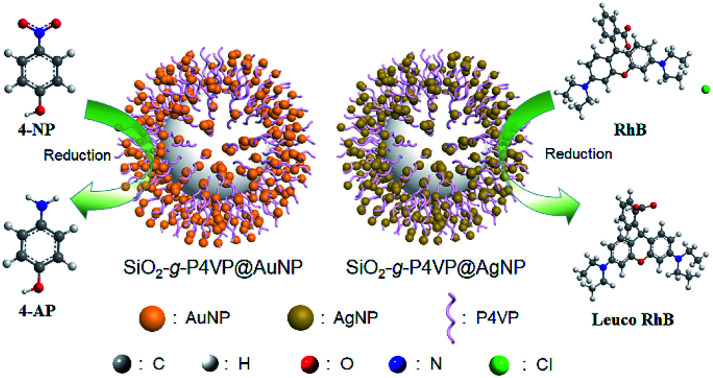

## 1 Introduction

Organic dyes, a major class of synthetic organic compounds, have been heavily used in agriculture and industry. Accordingly, a large volume of wastewater containing various organic dyes is produced. The inevitable release of organic dyes represents a critical issue, posing a serious environmental problem and a public health risk.^1^ Concerning the non-biodegradability and good water solubility of many widely applied organic dyes, it is absolutely necessary to remove these organic contaminants from water resources because of their intrinsic toxicity and carcinogenicity.^2,3^ During the past few decades, a variety of strategies, such as coagulation, chemical methods, electrochemical reduction and oxidation, microbiological treatments, enzymatic decompositions, and advanced oxidation processes have been developed to deal with these highly toxic and refractory organic pollutants.^4–11^ Of these approaches, degradation of organic dyes with catalytic materials has intrigued a great deal of attention because of its simplicity and high efficiency.^12–14^

Noble metal nanoparticles (MNPs) can be used as efficient catalysts, owing to their specific shapes, well-controlled small sizes, large surface-to-volume ratio, and different electronic properties.^15–19^ However, the direct utilization of MNPs in a liquid-catalysis process has been limited by their irreversible agglomeration and time-consuming separation.^20,21^ To address this issue, one of the most effective routes is immobilization of MNPs on solid supports such as polymers,^22–25^ mesoporous silica,^26–28^ metal–organic frameworks (MOFs),^29,30^ carbon,^31–34^ fibers,^35,36^ and metal oxides,^37,38^ which may improve their dispersity and stability.^39^ Among these nanostructures, silica nanospheres are deemed as good candidates for MNP catalyst supports due to their uniform spherical geometry, and good chemical stability.

Over the past few decades, much effort has been devoted to the deposition of MNPs onto the surface of silica spheres functionalized with carboxyl (–COOH),^40^ amino (–NH_2_),^41,42^ or mercapto (–SH) groups.^43^ However, dense decoration of MNPs on the silica spheres is difficult to achieve because full surface modification of these functional groups throughout the whole silica surface is often difficult. It was reported that polyelectrolyte brushes tethered on silica-based spheres (as called “hairy” particles) can serve as ideal support systems for the immobilization of small MNPs^44–47^ because the polyelectrolytes such as poly(4-vinylpyridine) (P4VP),^48–51^ polyacrylic acid (PAA),^52–55^ poly(2-(dimethylamino)ethyl methacrylate) (PDMAEMA),^56–58^ poly(dopamine) (PDA),^59–61^ polyamidoamine (PAMAM),^62,63^ possessing abundant functional groups have strong binding affinity to metal particles or the parent precursor metal ions. Among the polyelectrolytes, P4VP with a unique pH-dependent protonation–deprotonation feature can act as good chelators to achieve efficient confinement of agglomeration-free MNPs.^64^

In this context, we describe herein an effective method for the fabrication of heterogeneous MNP catalysts supported on hairy silica spheres and their efficiency in promoting the degradation of organic dyes. The P4VP brushes were covalently tethered onto the surface of SiO_2_ nanospheres through surface-initiated atom transfer radical polymerization (SI-ATRP), followed by *in situ* growth of tiny highly dispersed MNPs in the P4VP layer, resulting in the hierarchical SiO_2_@P4VP/MNP structure. The covalently grafted P4VP brushes on the surface of SiO_2_ may lead to the objective of dispersing in aqueous solution and thus circumvent the aggregation and leaching of MNPs. Furthermore, these hierarchical SiO_2_@P4VP/MNP composites could be employed as heterogeneous nanocatalysts for the degradation of organic dyes with good reusability.

## 2 Experimental section

### 2.1 Materials

Chloroauric acid (HAuCl_4_·3H_2_O, 99.9%), silver nitrate (AgNO_3_, AR grade), 4-vinylpyridine (4VP, 98%), tris[2-(dimethylamino)ethyl]amine (Me_6_TREN, 99%), copper(i) bromide (CuBr, 98%), 4-nitrophenol (4-NP, 99.5%), Rhodamine B(RhB, 98%) and sodium borohydride (NaBH_4_, 98%) were purchased from Sigma-Aldrich. Tetraethyl orthosilicate (TEOS, 98%) and trichloro-[4-(chloromethyl)phenyl]silane (97%) were supplied from Alfa Aesar. Technical-grade ethanol, *N*,*N*-dimethylformamide (DMF) and anhydrous tetrahydrofuran (THF) were purchased from WANQING Chemical Glass ware and Instrument Co., Ltd. (Nanjing, China).

### 2.2 Characterization

Transmission electron microscopy (TEM) was performed on a JEM-1400 (JEOL) operated at 100 kV. The samples were prepared by dropping several droplets of the nanoparticle solution on the carbon coated copper TEM grids and allowed to dry in air. Fourier-transform infrared (FTIR) spectra were recorded by using a Magna-550 Fourier transform infrared spectrometer. Thermogravimetric analysis (TGA) was performed under air flow from 20 to 800 °C in a Dupont 651US system. The heating rate of TGA was 20 °C min^−1^. Ultraviolet-visible (UV-vis) absorption spectra of the samples were measured at room temperature on a Lambda 750 UV-vis spectrophotometer. Powder X-ray diffraction (XRD) patterns were recorded on a Siemens D5005 diffractometer.

### 2.3 Synthesis of initiator-immobilized SiO_2_ nanospheres (*i.e.*, SiO_2_–Cl)

Firstly, bare SiO_2_ nanospheres with a diameter of *ca.* 330 nm were synthesized using the Stöber method with a little modification.^65^ In a typical synthetic route, 3.9 mL of TEOS was treated with 5.6 mL of 25% ammonia (aq.), 60 mL of ethanol and 22 mL of water. The product was isolated by centrifugation after stirring at room temperature for 24 h. The sediments were washed with anhydrous tetrahydrofuran (THF) three times and then dried at 45 °C under vacuum. Subsequently, 1.0 g of dried SiO_2_ nanospheres was suspended in 10 mL of anhydrous THF in a flask which was degassed with Ar for 20 min. Then, 1.5 g of trichloro-[4-(chloromethyl)phenyl]silane was added dropwise to the stirring suspension under an Ar atmosphere. The surface immobilization was carried out for another 24 h. The initiator-immobilized SiO_2_ nanospheres were isolated by centrifugation with three cycles of ethanol/THF rinsing, followed by drying under vacuum at room temperature.

### 2.4 Synthesis of P4VP-grafted SiO_2_ spheres (*i.e.*, SiO_2_-*g*-P4VP)

100 mg of SiO_2_–Cl, 5 mL of 4VP, 5 mL of *N*,*N*-dimethylformamide (DMF), 17.5 mg of CuBr and 50 mg of Me_6_TREN were vacuumed by three freeze-thaw-cycles in liquid argon. The mixture was stirred at 50 °C for 48 h. Then, the ampule was taken out from the oil bath to terminate the polymerization. The product was precipitated from hexane and washed with 2-propanol to remove the P4VP homopolymer. The purification process was repeated three times. The resulting SiO_2_-*g*-P4VP was dried under vacuum at 40 °C for 24 h.

### 2.5 Fabrication of SiO_2_-*g*-P4VP/MNP composites

10 mg of SiO_2_-*g*-P4VP was dispersed in 10 mL of DMF. 1 mL of HAuCl_4_ or AgNO_3_ (10 mM) was then added to the above solution and vigorously stirred for 0.5 h. Then, 1 mL of NaBH_4_ solution (10 mM) was added with stirring for 1 h. The resulting SiO_2_-*g*-P4VP/MNP composites were isolated by centrifugation at 6000 rpm for 5 min, then washed with water several times and dried under vacuum at 40 °C for 24 h.

### 2.6 Reductive degradation of 4-nitrophenol using SiO_2_-*g*-P4VP/AuNPs

Typically, 1.0 mg of SiO_2_-*g*-P4VP/AuNP composites was dispersed in 15 mL of 4-NP aqueous solution (0.125 mM). Then, 8 mL of fresh NaBH_4_ solution (10 mM) was added to the mixture for the reduction reaction. 1 mL of the solution mixture was taken and centrifuged for the determination with UV-vis absorption spectra at 3 min intervals.

### 2.7 Reductive degradation of rhodamine B using SiO_2_-*g*-P4VP/AgNPs

Typically, 1.0 mg of SiO_2_-*g*-P4VP/AgNP hybrids was dispersed into 10 mL of RhB aqueous solution (0.03 mM). Then, 8 mL of fresh NaBH_4_ solution (4 mM) was added to the mixture for the reduction reaction. 1 mL of the solution mixture was taken and centrifuged for the determination with UV-vis absorption spectra at 2 min intervals.

## 3 Results and discussion

### 3.1 Fabrication and characterization of SiO_2_-*g*-P4VP/MNP nanocatalysts

**Fig. 1:**
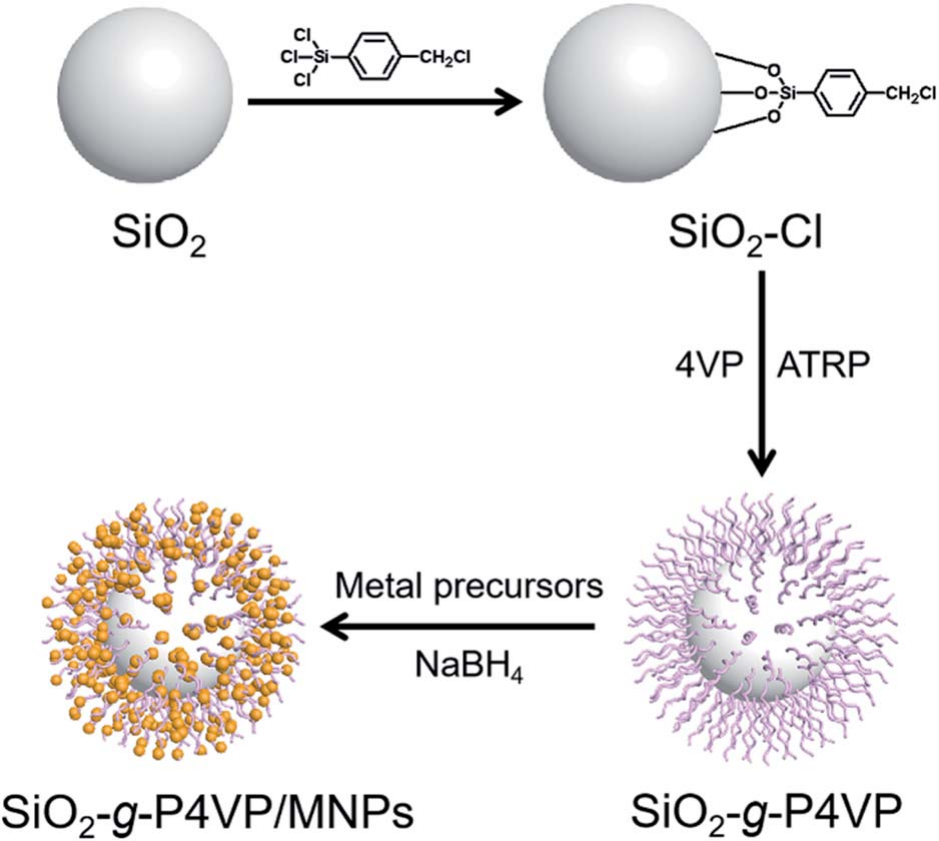
Schematic illustration of the fabrication of SiO_2_-*g*-P4VP/MNPs in which MNPs are embedded in the hairy P4VP matrix on the silica core.

The synthetic procedure for the preparation of the SiO_2_-*g*-P4VP/MNP hybrid nanocomposite is illustrated in Fig. 1. Bare silica nanospheres with rich Si–OH groups were prepared using the Stöber method.^65^ To functionalize the surface of the silica nanospheres with a benzyl chloride initiator, an excess amount of a silane coupling agent (*i.e.*, trichloro(4-chloromethylphenyl) silane) was added. The esterification reaction between trichloro(4-chloromethylphenyl) silane and hydroxyl groups on the surface of SiO_2_ nanospheres generated the macroinitiator (*i.e.*, SiO_2_–Cl). The grafting of P4VP from silica nanospheres was achieved by surface-initiated ATRP of 4VP, producing the hairy silica spheres (*i.e.*, SiO_2_-*g*-P4VP). P4VP, a functional polymer, exhibits excellent binding ability to many cations, such as H^+^, Ag^+^, Au^3+^, and Pd^2+^.^66^ Therefore, the obtained P4VP-grafted silica nanospheres can serve as good chelators, which coordinate with metal precursors (*i.e.*, AuCl_4_^−^, Ag^+^) to form metal ion complexes. Well-dispersed small MNPs were grown on the surface of SiO_2_ nanospheres by reduction of the corresponding metal precursors using NaBH_4_ as a reducer, leading to the formation of SiO_2_-*g*-P4VP/MNP nanocomposite.

**Fig. 2:**
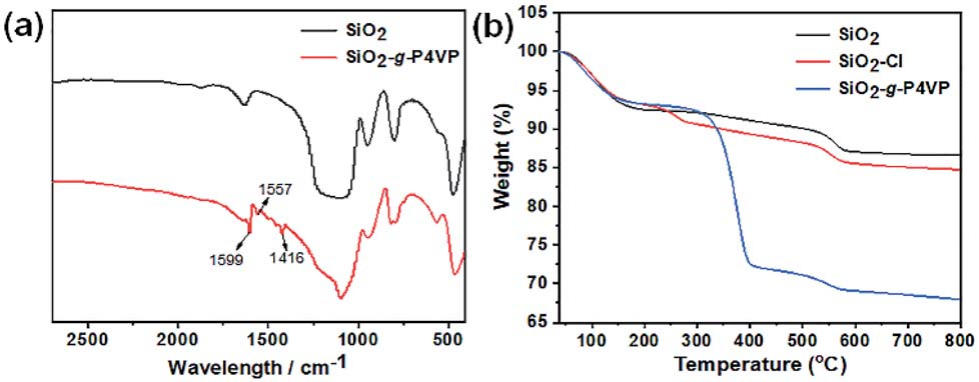
(a) FTIR spectra of bare silica nanospheres and SiO_2_-*g*-P4VP core–shell nanoparticles. (b) TGA curves of SiO_2_ nanospheres, SiO_2_–Cl and SiO_2_-*g*-P4VP.

Fourier transformed infrared (FTIR) spectra were measured to probe the structure of the hairy silica nanospheres. As illustrated in Fig. 2a, the strong absorption bands at 1087 cm^−1^ for both bare silica and SiO_2_-*g*-P4VP were assigned to the asymmetric stretching vibrations of Si–O–Si bonds. After ATRP of 4VP, three newly emerged peaks at 1599, 1557 and 1416 cm^−1^ were observed clearly. They corresponded to the stretching vibrations of pyridyl rings, which confirmed the successful grafting of P4VP onto the silica surface. Thermogravimetry analysis (TGA) was also used to determine the composition of the samples (Fig. 2b). The weight loss of bare SiO_2_, SiO_2_–Cl, and SiO_2_-*g*-P4VP was about 6 wt% at 150 °C, possibly attributed to the physically absorbed water and other residual organic species. There was ∼2 wt% of difference in the weight loss at about 250 °C between bare (*i.e.*, SiO_2_) and silane coupling agent-functionalized silica nanospheres (*i.e.*, SiO_2_–Cl), which was attributed to the thermal decomposition of trichloro(4-chloromethylphe-nyl) silane. The dramatic difference in the weight loss up to 32 wt% between SiO_2_–Cl and SiO_2_-*g*-P4VP could be mainly ascribed to combustion of the grafted P4VP chains, confirming the successful grafting of P4VP from the SiO_2_ nanospheres. And the weight component of the P4VP shell on the silica core was calculated to be 30 wt%.

**Fig. 3:**
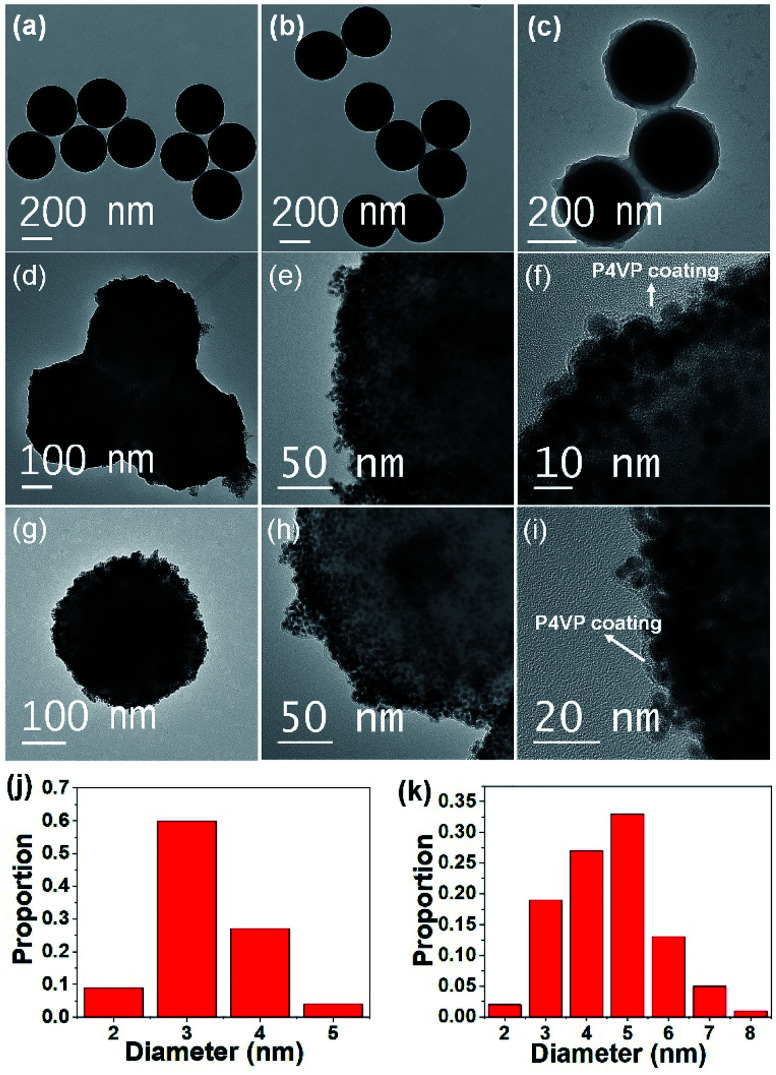
TEM images of (a) bare silica nanospheres, (b) SiO_2_–Cl, (c) SiO_2_-*g*-P4VP, (d) SiO_2_-*g*-P4VP/AuNPs and (g) SiO_2_-*g*-P4VP/AgNPs. HRTEM images of (e and f) SiO_2_-*g*-P4VP/AuNPs and (h and i) SiO_2_-*g*-P4VP/AgNPs. Size distribution histograms of MNPs on (j) SiO_2_-*g*-P4VP/AuNPs and (k) SiO_2_-*g*-P4VP/AgNPs, corresponding to TEM images in (d) and (g), respectively.

TEM was employed to perform the imaging of the materials in each step. The TEM image of bare silica spheres showed smooth surfaces. Their sizes were uniform with an average diameter of ∼330 nm (Fig. 3a). Although their surfaces were functionalized with a silane coupling agent, there was no noticeable change observed in the TEM image (Fig. 3b). It is due to the negligible length of the organic species. After ATRP of 4VP, the light contrast of the hairy P4VP shell encompassing deep contrast of the silica core was clearly observed, verifying the P4VP-grafted silica core–shell structure (Fig. 3c). The thickness of the P4VP layer was ∼25 nm, determined by TEM analysis. Finally, Au or Ag NPs were deposited on the hairy P4VP-SiO_2_ support by *in situ* reduction of the corresponding metal precursors (*i.e.*, HAuCl_4_ or AgNO_3_) using NaBH_4_ as a reducing agent. The TEM micrographs in Fig. 3d, e, g and h show that the P4VP-grafted silica spheres were densely and homogeneously covered with small AuNPs and AgNPs, respectively. The low magnification TEM image depicts that the as-prepared SiO_2_-*g*-P4VP/MNP composites tended to closely pack on the TEM grid (Fig. S1†). In addition, the HRTEM image in Fig. 3f and i clearly shows that numerous Au or Ag NPs (the black dots) were incorporated into the P4VP matrix. In addition, no scattered MNPs were observed beyond the silica spheres in the TEM images. This reveals that the hairy P4VP chains make it efficient to catch the parent precursor metal ions through electrostatic attractions. And they were rapidly reduced to a mass of tiny MNPs that were uniformly distributed in the P4VP shell on the silica core.

It should be noted that the small MNPs are well stabilized by the P4VP chains which serve as protective coatings (marginal area with light contrast as marked in Fig. 3f and i), preventing the migration and aggregation of MNPs that are embedded in them. This stabilizing effect would play an important role in terms of durability and sustainability for catalytic applications. The size distribution histograms of Au and Ag NPs deposited on the hairy silica spheres are depicted in Fig. 3j and k. Calculated from the statistical data of 100 MNP numbers, the average size of the Au NPs and Ag NPs was ∼3.3 and ∼4.5 nm, respectively. The highly dispersed and small-sized MNPs immobilized on the silica surface will be conducive to improving catalytic activities since MNPs with smaller particle sizes have a larger surface-to-volume ratio to provide more exposed metal atoms which may serve as the potential catalytic sites.

**Fig. 4:**
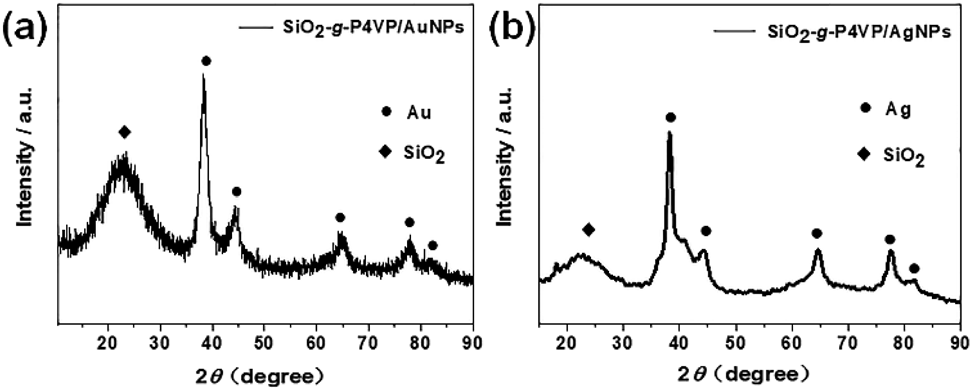
XRD patterns of (a) SiO_2_-*g*-P4VP/AuNPs and (b) SiO_2_-*g*-P4VP/AgNPs.

The phase of the SiO_2_-*g*-P4VP/MNP composites is examined by wide-angle XRD (Fig. 4). A typical XRD pattern of the obtained SiO_2_-*g*-P4VP/AuNPs is shown in Fig. 4a. The broad peak at 2*θ* of 22° corresponded to amorphous silica. Five diffraction peaks at 2θ of 38.20°, 44.19°, 64.32°, 78.01° and 82.16° were due to the reflection from the crystalline planes of (111), (200), (220), (311) and (222), which is in good accordance with standard facecentered-cubic (fcc) gold (JCPDS file: 04-0784),^67^ indicating that highly crystalline Au NPs formed in the P4VP matrix. A typical XRD pattern of the obtained SiO_2_-*g*-P4VP/AgNPs is shown in Fig. 4b. The obvious diffraction peaks at 2*θ* of 38.24°, 44.34°, 64.69°, 77.55° and 81.88° can be readily indexed to the fcc structure of Ag, and shows good agreement with the standard data (JCPDS file: 04-0783).^68^ The broad peak centered at 22° corresponding to SiO_2_ was also observed.

### 3.2 Catalytic activity of SiO_2_-*g*-P4VP/MNPs toward the degradation of organic dyes

**Fig. 5:**
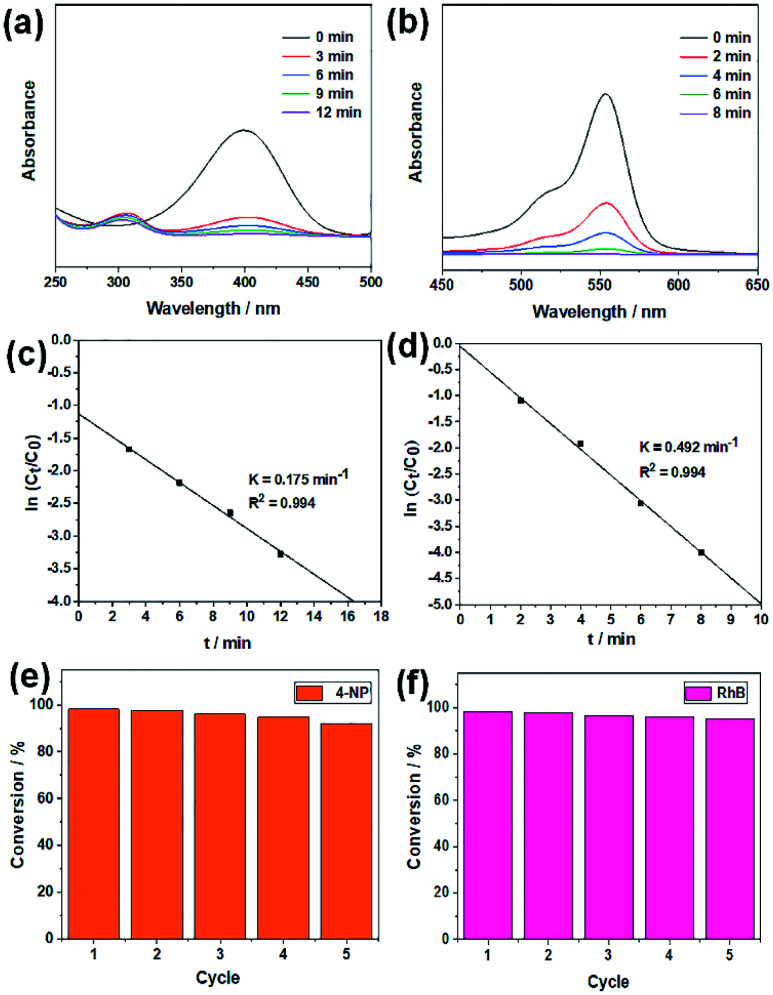
Time-dependent UV-vis spectral monitoring of the reductive degradation of (a) 4-NP using SiO_2_-*g*-P4VP/AuNPs as a catalyst at 3 min intervals and (b) RhB using SiO_2_-*g*-P4VP/AgNPs as a catalyst at 2 min intervals. Plots of ln(*C*_*t*_/*C*_0_) *vs.* reaction time for the reductive degradation of (c) 4-NP and (d) RhB. The reusability of (e) SiO_2_-*g*-P4VP/AuNPs for the reduction of 4-NP and (f) SiO_2_-*g*-P4VP/AgNPs for the reduction of RhB after five rounds of cycling.

Two organic dyes, *i.e.*, 4-nitrophenol (4-NP) and rhodamine B (RhB) are monitored for chemical decolorization by the hairy silica/MNP composites. 4-NP is a toxic organic pollutant, but its reduced form, *i.e.*, 4-aminophenol (4-AP), is very useful in medicinal applications such as analgesic and antipyretic drugs. The reductive degradation of 4-NP to 4-AP in the presence of NaBH4 was investigated to evaluate the catalytic activity of SiO_2_-*g*-P4VP/AuNPs. As shown in Fig. 5a, the principal absorption peak of 4-NP at 400 nm significantly decreased while that at 300 nm gradually increased upon the addition of 1.0 mg of SiO_2_-*g*-P4VP/AuNPs as a catalyst in the presence of NaBH_4_, showing good agreement with previously reported results. Simultaneously, the color gradually changed from yellow to colorless (Fig. S2a†), indicating the transformation of 4-NP to 4-AP. And the reduction reaction was finished within 12 min. RhB, another common organic dye in effluents of the ink-manufacturing process, was selected as a model pollutant to assess the catalytic activity of SiO_2_-*g*-P4VP/AgNPs. The main characteristic absorption band of RhB at 554 nm arises from the n → π transition of carbonyl (C=O) and imine (C=N) groups which contributed to its reddish-violet color and decreased gradually to a value of zero (Fig. 5b). All RhB was completely decomposed in 8 min, as the solution turned from reddish-violet to totally colorless (Fig. S2b†).

The kinetic plots of ln(*C*_*t*_/*C*_0_) *versus* reaction time for the two catalytic reactions are shown in Fig. 5c and d. A linear relationship between ln(*C*_*t*_/*C*_0_) values and reaction time was obtained in these two catalytic reactions, indicating that the reactions follow the pseudo-first-order kinetics. Therefore, it can be described as a first-order linear relationship ln(*C*_*t*_/*C*_0_) = −*kt*, where *C*_0_ and *C*_*t*_ are the concentrations of the dyes at the beginning and time *t* of the reaction, respectively, and *k* is the first-order rate constant. The apparent rate constants (*k*_app_) in the reductive degradation of 4-NP and RhB are calculated to be 0.175 and 0.492 min^−1^ using SiO_2_-*g*-P4VP/AuNPs and SiO_2_-*g*-P4VP/AgNPs as heterogeneous catalysts, respectively. We also compared the catalytic activities of SiO_2_-*g*-P4VP/MNP nanocatalysts with various heterogeneous catalysts reported in other published studies. As shown in Table 1, the catalytic activities of SiO_2_-*g*-P4VP/MNPs in the reductive degradation of 4-NP and RhB were superior to the previously reported heterogeneous nanocatalysts which have been used in this field. The dye pollutant removal efficiency using SiO_2_-*g*-P4VP/MNPs as heterogeneous catalysts was also compared with the previously reported literature (Table S1†). It is notable that this work presents a superior dye removal efficiency compared to the published studies.

**Table 1:** Comparison of the catalytic activities of SiO_2_-*g*-P4VP/MNPs with other heterogeneous catalysts in the literature for the reductive degradation of 4-NP and RhB

Dyes	Nanocatalysts	Size (nm)	Rate constant [min^−1^]	Reference
4-NP	Au/graphene hydrogel	8–25	0.19	69
	PNIPAM-*b*-P4VP-Au^a^	3.3	0.09	70
	GO/CNT-Au^b^	4.1	0.160	71
	Au/C^c^	4.6	0.106	72
	SiO_2_-*g*-P4VP/AuNPs	3.3	0.175	This work
RhB	Ag@Cu bimetallic NPs	4–32	0.366	73
	FeAgPt alloy NPs	10–20	0.256	74
	ZnO/AgNPs	2–6	0.0296	75
	Ag@ZBW^d^	16.7	0.02	76
	Fe_3_O_4_@ SiO_2_@Ag	10–20	0.15	77
	SiO_2_-*g*-P4VP/AgNPs	4.5	0.492	This work

a Poly(*N*-isopropylacrylamide)-*b*-poly(4-vinyl pyridine).

b Graphene oxide/carbon nanotube–gold composites.

c Sintered carbon-supported gold nanocatalyst.

d AgNPs@ZnO nanorods@Bi_2_WO_6_.

Despite the noticeable catalytic capability of the SiO_2_-*g*-P4VP/MNPs, it would actually not be suitable for wastewater treatment on a large scale in practice unless it has good reusability. Thus, the cyclic stability of SiO_2_-*g*-P4VP/MNPs is assessed during successive cycles of the reduction reaction, as depicted in Fig. 5e and f. The nanocatalysts were recovered by centrifugation at 6000 rpm for 5 min. After five cycles of recycling, the reduction efficiency of SiO_2_-*g*-P4VP/AuNPs reached about 92.2% (Fig. 5e), and that of SiO_2_-*g*-P4VP/AgNPs is 95.2% (Fig. 5f). These results indicate there is no obvious loss of catalytic activities of SiO_2_-*g*-P4VP/MNPs upon recycling up to five rounds. In addition, after the fifth recycling, the SiO_2_-*g*-P4VP/MNP nanocatalysts were further characterized by TEM. As shown in Fig. S3,† no obvious aggregation or leaching of MNPs on the hairy silica spheres was found in the TEM observations, indicating that the covalently grafted P4VP brushes on the silica surface can prevent the coagulation and dissociation of MNPs in the catalytic reaction.

**Fig. 6:**
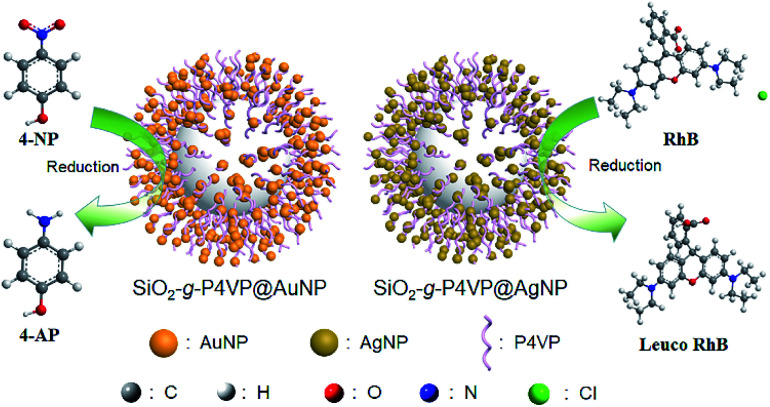
Schematic illustration of the reductive degradation of 4-NP and RhB using SiO_2_-*g*-P4VP/MNPs as heterogeneous catalysts.

Fig. 6 shows the reductive degradation process of 4-NP and RhB employing SiO_2_-*g*-P4VP/MNPs as heterogeneous catalysts. In the MNP-catalyzed reduction process, the electron transfer occurred from the donor (*i.e.*, NaBH_4_) to the acceptor (*i.e.*, 4-NP and RhB) on the surface of MNPs.^78^ The detailed catalytic mechanisms for the reductive degradation with excess NaBH_4_ are depicted in Fig. S4 and S5.† In the case of 4-NP, firstly, the hydride ions in NaBH_4_ adsorb onto the AuNP surface to form the gold hydride species. Then, the hydride transfers from the gold hydride species to 4-nitrophenol (1), leading to the reduction of 4-NP into nitroso intermediate 2, 3, and further to 4-nitrosophenol (4). The intermediate 4 is then reduced by the hydride transfer from the gold hydride species to intermediate 5, and further to corresponding stable hydroxyamino 6. Finally, the intermediate 4-(hydroxyamino)phenol (6) is reduced to 4-aminophenol (7) by further hydride transfer (Fig. S4†). In the case of RhB, the nucleophile BH_4_^−^ anion donates an electron to AgNPs on the hairy silica spheres, and the electrophilic RhB cation (1) would capture the electron from AgNPs to generate intermediate 2. The rearrangement of the electrons in intermediate 2*via* aromatization produces zwitterion 3, followed by the cyclization reaction to yield the leuco RhB (4) (Fig. S5†). As demonstrated in the reaction mechanisms, the MNPs serve as an electron transporting medium. And we conducted a blank control test for the degradation of 4-NP and RhB by SiO_2_-*g*-P4VP in the presence of NaBH_4_ for comparison, which was monitored using UV-vis spectra (Fig. S6†). Without the decorated MNPs on the hairy silica spheres (*i.e.*, SiO_2_-*g*-P4VP), the organic dyes hardly degraded.

The good catalytic activities of the SiO_2_-*g*-P4VP/MNP nanocomposites are derived from the highly dispersed and small-sized MNPs on the silica support and the potential long-range charge transfer effect. Recently, Nørskov *et al.* reported that the adsorbates are more strongly adsorbed on metal oxide supported by noble metals as conductive substrates rather than the pure metal oxide system due to the significant long-range charge transfer effect to the oxygen-containing adsorbates.^79^ We speculated that there was an improved electron charge transfer from the heterogeneous catalyst to the adsorbed organic dyes (*i.e.*, oxygen-containing species), induced by the surface-immobilized MNPs on the hairy silica spheres. More importantly, the P4VP brushes on the silica surface also play a crucial role in facilitating the reduction reaction. On the one hand, the pyridyl groups of P4VP tethered on SiO_2_ can interact with the phenolic groups of 4-NP to form hydrogen bonding. These hydrogen-bonding interactions increase the chances for random adsorption of 4-NP on the silica surface where there are a large number of AuNP nanocatalysts. Thus, the P4VP promotes the migration of 4-NP from solution to the surroundings of AuNPs. On the other hand, NaBH_4_ adsorbed onto the AuNP surface would act as a hydrogen supplier to attack electron acceptor RhB, leading to its reduction. In the case of RhB, P4VP bearing abundant pyridine moieties has excellent binding ability to cations.^66^ It is beneficial to the adsorption of cationic RhB *via* electrostatic interaction, which facilitates the transfer of electrons from NaBH_4_ to the cationic RhB on the surface of AgNPs, thus greatly increasing the catalytic efficiency. It could be concluded that the interactions between P4VP chains and organic dyes significantly enhance the adsorption of dyes onto the catalyst surface and therefore contribute to an increased degradation rate. The adsorption experiments of organic dyes on SiO_2_-*g*-P4VP were carried out to verify the important role of P4VP in the reaction. The organic dyes in aqueous solution were added to SiO_2_-*g*-P4VP and mixed well by vortexing. The suspension was incubated at room temperature for 2 h and then centrifuged. The absorbance of the supernatant was recorded by UV-vis spectroscopy, as shown in Fig. S7.† The absorption band decreased obviously as the amount of SiO_2_-*g*-P4VP increased, indicating the strong absorbing ability of SiO_2_-*g*-P4VP. This favorable adsorption of organic dyes to the surface of SiO_2_-*g*-P4VP provides a high dye concentration environment around the nanocatalyst, which can promote the reductive degradation rate.

## 4 Conclusion

In summary, we demonstrated a facile and effective route to prepare highly dispersed small MNPs with a narrow size distribution on the hairy P4VP-SiO_2_ support. The as-prepared SiO_2_-*g*-P4VP/MNP composites serving as recyclable heterogeneous catalysts can efficiently catalyze the reduction of 4-NP and RhB dyes. A number of advantageous features of these heterogeneous catalysts for the reductive degradation of organic dyes could be concluded: (1) small-sized and highly dispersed MNPs immobilized on the silica surface for enhanced catalytic activity, (2) P4VP coating for the protection of the small MNPs from aggregations during the catalytic reaction, (3) interactions between P4VP chains and organic dyes significantly enhance dye adsorption and thus accelerate the catalytic reduction rate. Doubtlessly, the hairy P4VP-SiO_2_ support can be extended to many other metals (*e.g.*, Co, Pd, Ru, Pt, Ni, *etc.*) towards the development of efficient and reliable heterogeneous catalysts for industrial applications.

## Author contributions

Xin Chen: data curation, formal analysis, visualization, and writing – original draft; Li Zhang: formal analysis and investigation; Bin Xu: methodology and funding acquisition; Tingting Chen and Lianhong Hu: investigation; Wei Yao and Mengxiang Zhou: validation; Hui Xu: conceptualization, supervision, funding acquisition, and writing – review & editing.

## Conflicts of interest

There are no conflicts to declare.

## Supplementary Material

NA-003-D1NA00020A-s001
