# Supplementary material for: Hairy silica nanosphere supported metal nanoparticles for reductive degradation of dye pollutants
Source: Nanoscale Adv. 2021 Mar 22;3(10):2879–86. doi: 10.1039/d1na00020a (PMC9419623; doi:10.1039/d1na00020a)
Supplement: NA-003-D1NA00020A-s001 [file NA-003-D1NA00020A-s001.pdf]

Electronic Supplementary Material (ESI) for Nanoscale Advances.
This journal is © The Royal Society of Chemistry 2021

Supporting Information

Hairy silica nanospheres supported metal nanoparticles for reductive degradation of dye pollutants

Xin Chen,[a] *Li Zhang ,*[a] *Bin Xu,*[b] *Tingting Chen,*[a] *Lianhong Hu,*[a] *Wei Yao,*[a] *Mengxiang Zhou,*[a] *and Hui Xu**[a]

[a]Institute of Advanced Synthesis, School of Chemistry and Molecular Engineering, Jiangsu National Synergetic Innovation Center for Advanced Materials, Nanjing Tech University, Nanjing 211816, China.

[b]Nanjing Institute of Environmental Sciences, Ministry of Ecology and Environment of the People's Republic of China, Nanjing 210042, China.

*Correspondence to E-mail: ias_hxu@njtech.edu.cn.

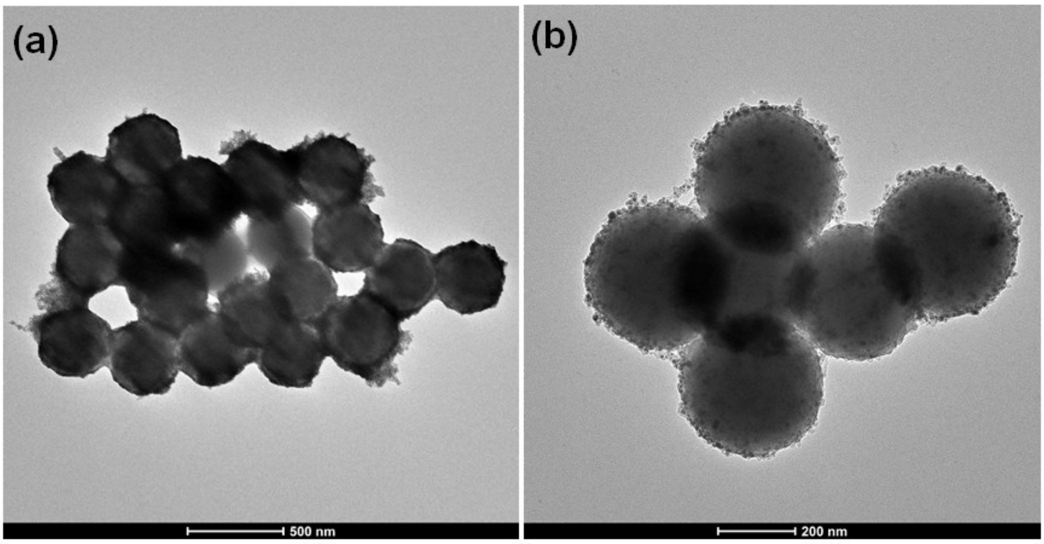

Figure S1. Low magnification TEM images of the as-prepared (a) SiO_2-*g*-P4VP/AuNP and (b) SiO_2-*g*-P4VP/AgNP composites.

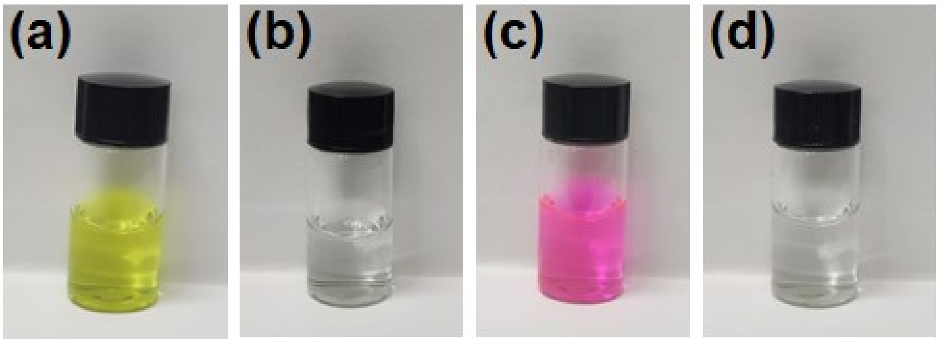

Figure S2. Solutions of 4-NP (a) before and (b) after reduction, RhB (c) before and (d) after reduction.

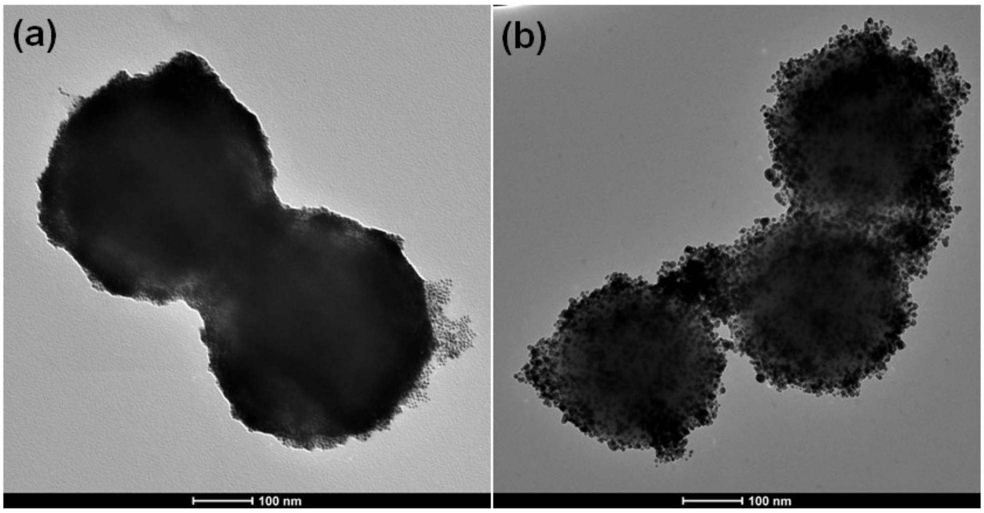

Figure S3. TEM images of (a) SiO_2-*g*-P4VP/AuNPs and (b) SiO_2-*g*-P4VP/AgNPs composites after the fifth recycling.

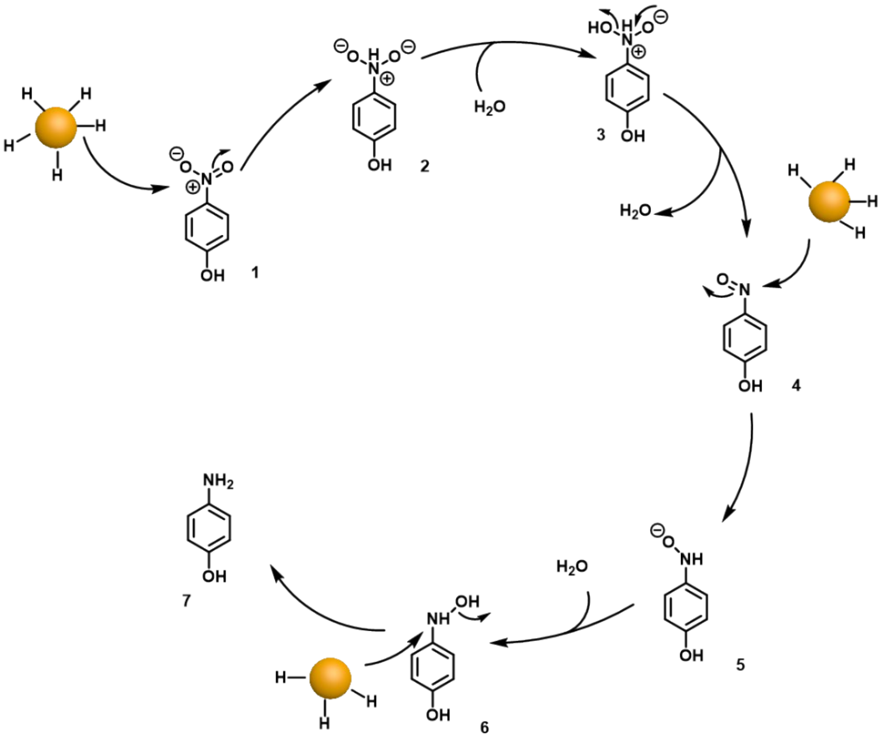

Figure S4. Proposed mechanism for the reduction of 4-nitrophenol catalyzed by SiO_2-*g*-P4VP/AuNPs.

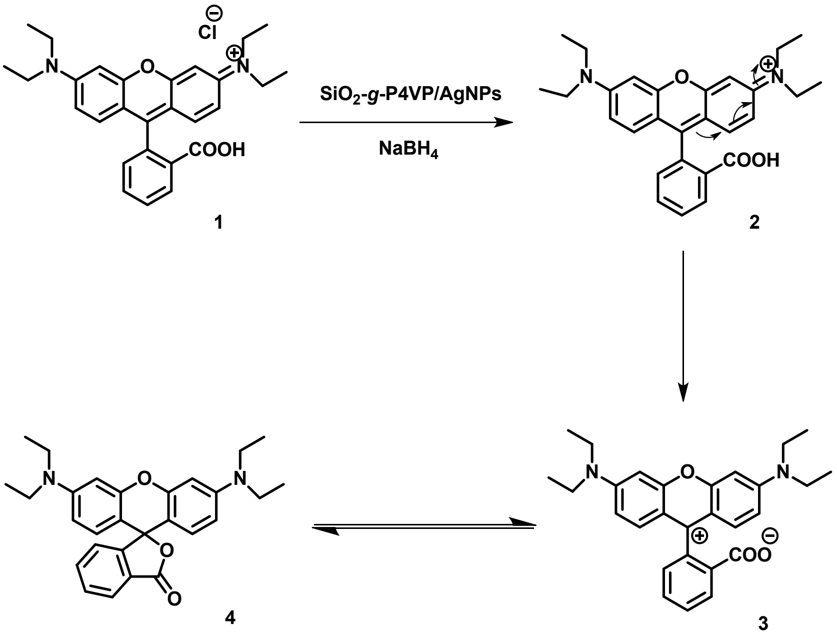

Figure S5. Proposed mechanism for the reduction of RhB catalyzed by SiO_2-*g*-P4VP/AgNPs.

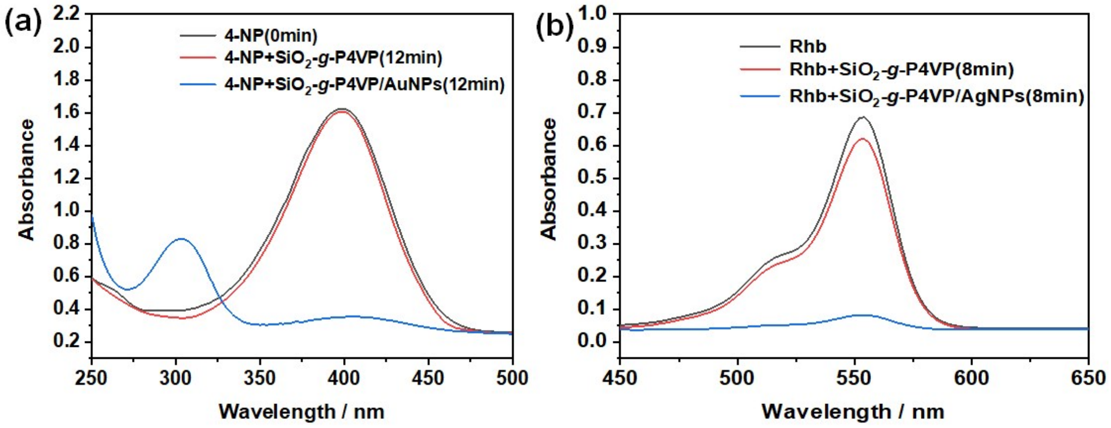

Figure S6. The degradation of organic dyes (a) 4-NP and (b) RhB for SiO_2-*g*-P4VP in the presence of $NaBH_4$ as the blank control test.

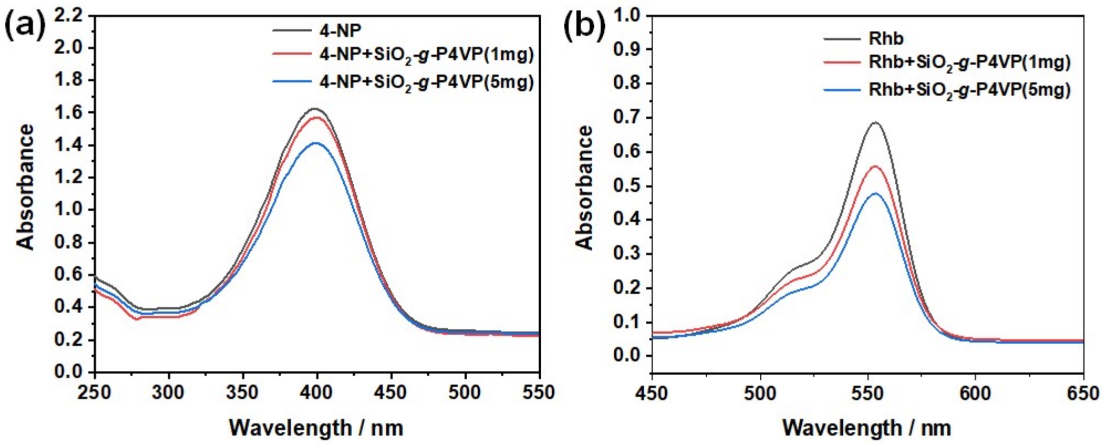

Figure S7. The adsorption experiments of organic dyes (a) 4-NP and (b) RhB on SiO_2-*g*-P4VP examined by UV−vis spectra monitoring. 10 mL of organic dyes (0.02 mg/mL) in aqueous solution was added with 1 or 5 mg of SiO_2-*g*-P4VP and mixed well by a vortex mixer. The suspension was incubated at room temperature for 2 h and then centrifuged at 6000 rpm for 10 min.

Table S1. Dye pollutant removal efficiency for the reductive degradation by compared with MNPs by different supports in previously reported works.

Dyes	Nanocatalysts	Dye removal efficiency[a]	Reference
4-NP[b]	Au@ porous SiO_2	~40%	1
	SiO_2@PDMAEMA–Au	~20%	2
	SiO_2-*g*-P4VP/AuNPs	92.7%	This work
RhB[c]	RGO/Ag	~50%	3
	SiO_2/Ag	~10%	4
	SiO_2-*g*-P4VP/AgNPs	99.4%	This work

[a]The dye decolorization was monitored by UV–vis absorbance and the dye pollutant removal efficiency was calculated using the following formula:

$$\text{Dye removal efficiency (\%)} = \frac{A_0 - A_t}{A_0} \times 100$$

where A_0 is the absorbance before decolorization, A_t is the absorbance after certain time *t* of dye removal.

[b]t =10 min, [c]t = 8 min.

Table S2. ICP characterizations of the SiO_2-*g*-P4VP/MNPs nanocatalysts before and after the reaction.

Catalyst	SiO_2-*g*-P4VP/MNPs	SiO_2-*g*-P4VP/MNPs (5 cycles later)
Au(wt%)	14.6%	13.7%
Ag(wt%)	6.9%	6.2%

Reference

1. Z. Wang, H. Fu, D. Han and F. Gu, The effects of Au species and surfactant on the catalytic reduction of 4-nitrophenol by Au@SiO_2, *J. Mater. Chem. A*, 2014, **2**, 20374-20381.
2. J. Chen, P. Xiao, J. Gu, D. Han, J. Zhang, A. Sun, W. Wang and T. Chen, A smart hybrid system of Au nanoparticle immobilized PDMAEMA brushes for thermally adjustable catalysis, *Chem. Commun.*, 2014, **50**, 1212-1214.
3. K. S. Divya, A. Chandran, V. N. Reethu and S. Mathew, Enhanced photocatalytic performance of RGO/Ag nanocomposites produced via a facile microwave irradiation for the degradation of Rhodamine B in aqueous solution, *Appl. Surf. Sci.*, 2018, **444**, 811-818.
4. İ. Deveci and B. Mercimek, Performance of SiO_2/Ag Core/Shell particles in sonocatalalytic degradation of Rhodamine B, *Ultrason. Sonochem.*, 2019, **51**, 197-205.
